# Early Spectral Resolution Predicts Later Speech Recognition in Adult Cochlear Implant Recipients

**DOI:** 10.1002/lary.70383

**Published:** 2026-02-23

**Authors:** Katelyn A. Berg, Jillian B. Roberts, Michael Z. Burchesky, René H. Gifford

**Affiliations:** ^1^ Department of Otolaryngology—Head and Neck Surgery Vanderbilt University Medical Center Nashville Tennessee USA; ^2^ Department of Audiology, Hearts for Hearing Foundation Oklahoma City Oklahoma USA; ^3^ Department of Otolaryngology, Audiology Division University of Pittsburgh Medical Center Pittsburgh Pennsylvania USA

**Keywords:** cochlear implants, EasyQSMD, longitudinal outcomes, predictive value, spectral resolution, speech recognition

## Abstract

**Objectives:**

To investigate the trajectory of spectral resolution in adult cochlear implant (CI) recipients and determine whether early spectral resolution measures can predict later speech recognition outcomes.

**Methods:**

Prospective, longitudinal study of 531 postlingually deafened adult CI recipients. Spectral resolution (EasyQSMD) and speech recognition (CNC words, AzBio sentences in quiet and noise) were evaluated from activation through 1‐year post‐activation. Growth curve models were fit to EasyQSMD thresholds across all timepoints to generate mode‐based estimates of early spectral resolution at 1‐month post‐activation. These model‐derived early thresholds were then used to predict later speech recognition outcomes (best of 6‐months and 1‐year) through linear regression analyses.

**Results:**

EasyQSMD thresholds improved from activation to 1‐week post‐activation, then stabilized through the first year. Model‐derived early EasyQSMD thresholds significantly predicted later speech recognition outcomes, explaining 3%–8% of the variance in performance.

**Conclusion:**

Spectral resolution rapidly stabilizes, reaching asymptotic performance by 1‐week post‐activation. The EasyQSMD provides a non‐linguistic assessment tool that demonstrates significant predictive value for subsequent speech recognition outcomes. This early predictive capability enables the identification of poor performers during a critical window when speech recognition skills are still developing and the brain's neuroplasticity is at peak potential. Integrating spectral resolution assessment into early post‐activation clinical protocols could improve individualized realistic expectations counseling and guide targeted interventions—potentially leading to improved overall CI adoption rates. The brief administration time and independence from linguistic content make the EasyQSMD particularly valuable for multilingual populations and during the earliest stages of auditory rehabilitation post‐CI.

**Level of Evidence:**

3.

## Graphical abstract

In 531 ears from 498 adult cochlear implant recipients, spectral modulation detection (EasyQSMD) stabilized within 1 week post‐activation and significantly predicted later speech recognition outcomes. Early spectral resolution thresholds can identify potential poor performers during the critical first month, enabling earlier implementation of targeted interventions.

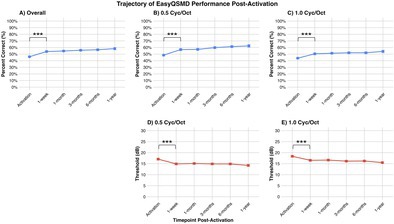

## 1 Introduction

Adult cochlear implant (CI) candidates frequently ask when they will understand speech post‐activation—a question clinicians struggle to answer precisely due to the variability in post‐activation speech recognition outcomes [1, 2, 3]. This uncertainty contributes to the alarmingly low CI utilization rates of just 2%–12% among eligible candidates [4, 5], as potential recipients hesitate to pursue surgery without clearer expectations [6]. Even after implantation, unmanaged expectations can lead to significant dissatisfaction, with recent data showing that up to 42% of adults express some level of regret about their decision to receive a CI [7]. Developing more accurate early predictors of speech recognition outcomes would help address this critical gap in our ability to effectively counsel patients.

The most recent and third edition of the adult minimum speech test battery (MSTB‐3) provides standardized speech recognition measures to quantify CI recipients' progress post‐activation [8], including consonant nucleus consonant (CNC) word recognition [9], and AzBio sentence recognition [10] in quiet and multi‐talker babble noise. While these measures are valuable for assessing long‐term outcomes, they present three significant limitations for early outcome prediction. First, they require considerable auditory experience to be reliable predictors [11]. Second, they are language‐dependent, making them problematic for prelingually deafened individuals and non‐native speakers. Third, they are not typically administered until several weeks or months post‐activation [8]. Consequently, poor performers remain unidentified until several months after activation, often beyond the critical period of the first 6 months of use when performance is expected to most rapidly improve [12, 13, 14]. This delay potentially postpones interventions such as device re‐programming, auditory training, and expectations counseling.

Spectral resolution—the ability to detect changes in the frequency domain of sound—provides the theoretical foundation for speech recognition. Unlike speech recognition tasks that rely on high‐level linguistic and cognitive processes [15, 16], spectral resolution represents a more fundamental auditory capability that likely develops earlier in the auditory rehabilitation process [17]. Better spectral resolution allows listeners to distinguish between speech sounds with similar temporal but different spectral characteristics (e.g., vowels and nasals) [18, 19, 20, 21, 22, 23], while poorer spectral resolution forces listeners to rely more heavily on temporal cues and top‐down processing [24, 25, 26, 27, 28].

The extent to which CI recipients rely on top‐down processing varies across speech materials and listening conditions. For speech recognition in quiet where substantial linguistic context is available, CI recipients demonstrate greater reliance on semantic and syntactic information compared to listeners with normal hearing [15]. This top‐down processing becomes even more critical for speech recognition in noise and for materials with limited context such as isolated word or phoneme recognition [23, 24, 25, 26, 27, 28]. The heavy reliance on top‐down processing and linguistic knowledge in speech recognition assessments creates barriers for certain populations. For example, neurocognitive and neurolinguistic factors—including inhibition‐concentration abilities and working memory—significantly contribute to individual differences in CI recipients' speech recognition scores [15]. These limitations highlight the need for validated, non‐language based outcome measures that correlate strongly with speech understanding.

Spectral modulation detection (SMD) and spectral ripple discrimination (SRD) have emerged as ideal candidates because they assess spectral resolution without requiring linguistic knowledge. Both SMD and SRD measure spectral resolution, but more specifically, SMD measures a listener's sensitivity to spectral peaks and valleys in the amplitude spectrum, whereas SRD measures their ability to detect changes in the spectral ripple density of the sound. Importantly, there is a well‐documented positive relationship (*r* = 0.86–0.92) between SMD/SRD and word recognition in adult CI recipients [18, 19, 20, 21, 22, 23].

The quick SMD (QSMD [29])—now known as the EasyQSMD [30]—was developed to address the clinical need for a validated non‐language based outcome metric. CI listeners demonstrate a large range of abilities at low spectral modulation frequencies (0.25–0.5 cycles/octave (cyc/oct)), which most closely correlate with speech recognition [29], while often showing near‐normal thresholds at higher frequencies such as 1.0 cyc/oct [20]. The EasyQSMD uses both 1.0 cyc/oct and 0.5 cyc/oct to balance accessibility with clinical relevance and is highly correlated with word recognition [29, 30]. And, unlike traditional laboratory‐based measures, the EasyQSMD can be completed in under 5 min, does not require linguistic proficiency, and is available for free download.

Interestingly, Drennan and colleagues developed a similar clinic‐friendly SRD measure and investigated its relationship with speech recognition longitudinally in 10 newly implanted adults [31]. They found that while word recognition improved significantly from 1 to 12 months post‐activation, SRD performance plateaued by 1 month. This suggests that spectral resolution stabilizes faster than speech recognition and could potentially serve as an indicator of long‐term outcomes. However, this investigation was limited to just two post‐activation timepoints, a single speech recognition measure, and a small sample size.

Building on this, the current study aimed to (1) describe the trajectory of postoperative performance on EasyQSMD from activation through 1‐year post‐activation, and (2) determine whether early EasyQSMD thresholds can predict later speech recognition outcomes for CNC word recognition and AzBio sentences in quiet and noise. We hypothesized that adult CI recipients would reach asymptotic performance on EasyQSMD sooner than on speech recognition tasks, in alignment with Drennan and colleagues [31]. We further hypothesized that early EasyQSMD thresholds would significantly predict later speech recognition outcomes across test measures, providing valuable prognostic information in the critical early stages of auditory rehabilitation with the CI.

## 2 Methods

### 2.1 Participants

We analyzed data from 531 postlingually deafened adult ears (498 participants) implanted between August 2013 and December 2017 at a single large academic medical center. All participants were enrolled prospectively as part of a longitudinal clinical trial involving all adult CI candidates at the center. All participants were tested in accordance with the protocol approved by the Institutional Review Board (IRB).

Inclusion criteria required completion of the EasyQSMD at activation and at least one additional timepoint within the first year post‐activation. Participants represented three electrode types (straight, Mid‐Scala, and perimodiolar) from two CI manufacturers, Advanced Bionics and Cochlear Ltd. Straight electrode models included the Cochlear 24RE(ST), CI244, Hybrid‐L, and Advanced Bionics 1 J. Perimodiolar electrodes included the Cochlear 24RE(CA) and CI512. Participant demographics are summarized in Table 1.

**TABLE 1:** Participant demographics.

	Total *N* = 531 ears
Age at CI (years)	
Mean	65.1
Range	18.5–101.2
Biological sex	282 males/216 females
CI ear	274 right ears/257 left ears
Manufacturer	
Advanced Bionics	253 (48%)
Straight	12
Mid‐Scala	241
Cochlear Ltd.	278 (52%)
Straight	156
Perimodiolar	122

### 2.2 Protocol

#### 2.2.1 SMD

SMD was assessed using the EasyQSMD [30], administered longitudinally at six timepoints: initial activation, 1‐week, 1‐month, 3‐months, and 6‐months, and 1‐year post‐activation. The EasyQSMD employs the method of constant stimuli with spectral modulation depths of 16, 14, 13, 11, and 10 dB at both 0.5 and 1.0 cycles/octave (cyc/oct). These parameters were selected to encompass the typical performance of adult CI recipients while avoiding floor and ceiling effects. Testing was completed in the CI‐alone condition with the contralateral ear masked using 60–65 dB HL of speech noise via an insert earphone, if applicable; bilateral participants were tested using each CI separately.

Testing sessions included 60 trials (six trials per modulation depth and rate combination). Stimuli were generated in MATLAB version 8.2 (R2013b), calibrated to 60 dB SPL(A), and presented from a loudspeaker at 0° azimuth, 1 m from the participant in a single‐wall sound booth. In each trial, participants heard three sequential sounds each separated by a 400‐ms gap: two unmodulated noise bands (125–5600 Hz) and one target stimulus containing spectrally modulated noise. Participants identified which sound was different by selecting the corresponding button [1, 2, 3], or on a touchscreen interface. Standardized written instructions were provided on a laminated card in each sound booth, but no formal training was provided before test administration due to clinical time constraints.

Psychometric functions were plotted for each modulation rate (0.5 and 1.0 cyc/oct), and thresholds corresponding to 75% correct performance were extracted via logistic regression. Thresholds were used for primary analyses because they provide a more precise psychometric estimate of spectral resolution ability by incorporating performance across all modulation depths tested, reducing measurement error compared to raw percent correct scores. Lower thresholds (in dB) indicate better spectral resolution. Raw percent correct scores were also calculated for clinical reference.

#### 2.2.2 Speech Recognition

Speech recognition was evaluated using three standardized measures from adult MSTB [8]: CNC words [9], AzBio sentences in quiet [10], and AzBio sentences in noise using co‐located 20‐talker babble at a +5 dB signal‐to‐noise ratio (SNR). CNC words and AzBio sentences in quiet were tested at 1‐, 3‐, 6‐, and 12‐months post‐activation, while AzBio sentences in noise were tested at 3‐, 6‐, and 12‐months, as this more challenging task was not routinely administered earlier in the rehabilitation process when performance would likely be poor. Responses were scored as percent correct by the number of words correctly repeated. All stimuli were calibrated to 60 dB SPL (A‐weighted) and presented from a single loudspeaker positioned at 0° azimuth, 1 m from the participant. Sessions followed a fixed order determined by the clinic protocol: CNC words, AzBio in quiet, AzBio in noise, and then EasyQSMD.

### 2.3 Statistical Analyses

Analyses were conducted in R version 4.4.1 [32]. Due to skewness in some distributions, median values, interquartile ranges, and minimum/maximum values were reported for all measures across timepoints. To obtain estimates of early spectral resolution, we employed growth curve models using linear mixed‐effects regression via the lme4 and lmerTest packages in R. Separate models were fit for 0.5 and 1.0 cyc/oct thresholds, using all available data from six timepoints (activation, 1‐week, 1‐month, 3‐months, 6‐months, and 1‐year post‐activation). Time was log‐transformed [log(weeks + 1)] to account for the non‐linear trajectory of improvement, where performance improved rapidly in the first weeks before plateauing. Models included random intercepts and slopes for each participant, allowing for individual variation in both baseline and rate of change.

The 0.5 cyc/oct model included 960 observations from 306 participants (mean = 3.1 observations per participant). The 1.0 cyc/oct model included 855 observations from 295 participants (mean = 2.9 observations per participant). From these models, we extracted participant‐specific predicted thresholds at 1‐month post‐activation to represent early spectral resolution for subsequent predictive analyses. This approach offers several advantages over selecting the best score from discrete timepoints: (1) it uses substantially more data (239% more observations compared to selecting only 1‐week or 1‐month), (2) it accounts for individual trajectories of improvement, (3) it reduces measurement error through model‐based estimation, and (4) it properly handles missing data under the missing‐at‐random assumption.

For examining the trajectory of EasyQSMD performance across timepoints, we used the same mixed‐effects models described above. Post hoc pairwise comparisons employed Tukey's Honest Significant Different (HSD) adjustment for multiple comparisons. We did this using the emmeans package, controlling the family‐wise error rate at α=0.05 across all 15 pairwise comparisons (6 timepoints = 15 unique pairs).

For later speech recognition outcomes, we used the maximum (best) score from the 6‐months and 1‐year timepoints for each participant. This approach was selected rather than growth curve modeling because: (1) speech recognition was only assessed at 2–3 timepoints, providing insufficient data for reliable trajectory estimation, and (2) prior research demonstrates that speech scores typically plateau by 6‐months, suggesting the best score from this period reasonably represents asymptotic performance [11, 12, 13]. Linear regression analyses evaluated the predictive power of model‐derived early EasyQSMD thresholds (at 1‐month) for later speech recognition outcomes. Separate regression models were constructed using 0.5 cyc/oct thresholds, 1.0 cyc/oct thresholds, and the average of both as predictors, with CNC word scores, AzBio sentences in quiet, and AzBio sentences in noise as the respective outcome variables. *R* ^2^ values, unstandardized regression co‐efficients (β), and significance levels were calculated for each model.

Regression diagnostic checks confirmed that all models met assumptions for linear regression. Residuals were normally distributed (Shapiro–Wilk *p* > 0.10), with no evidence of heteroscedasticity (Breusch‐Pagan *p* > 0.15). No observation had Cook's distance > 0.5, indicating no single observation was excessively influential.

To evaluate whether missing data might bias our results, we examined baseline differences between participants with complete versus incomplete follow‐up data. Independent samples *t*‐tests comparing activation EasyQSMD thresholds showed no significant differences (0.5 cyc/oct: *t* = 0.42, *p* = 0.67; 1.0 cyc/oct: *t* = 0.91, *p* = 0.36), supporting the missing‐at‐random assumption underlying our analytical approach.

We conducted supplementary analyses to examine whether available demographic factors (age at implantation and biological sex) altered the predictive relationships between early spectral resolution and later speech outcomes. These co‐variates were added to the regression models described above. Participants with missing data were included using available case analysis, which utilized all non‐missing values for each measure and timepoint. This approach maximized statistical power but resulted in varying sample sizes across different measures and timepoints, reflecting common challenges in longitudinal data collection in clinical settings.

## 3 Results

### 3.1 Trajectory of Postoperative EasyQSMD Performance

**FIGURE 1:**
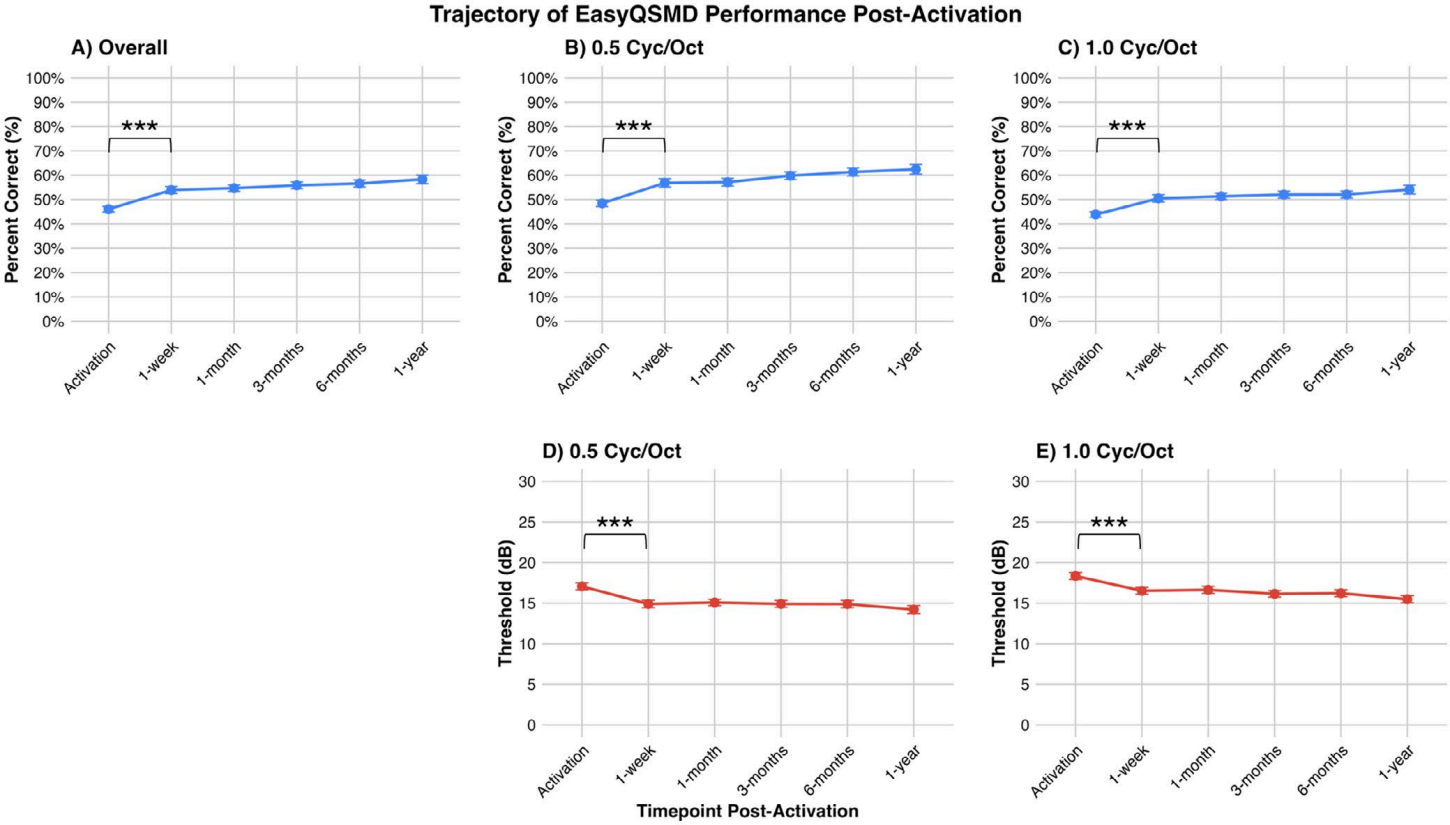
Mean and standard errors for EasyQSMD scores by each of the six timepoints: Activation, 1‐week, 1‐month, 3‐months, 6‐months, and 1‐year post‐activation. The top row shows scores in percent correct (%) for (A) overall, (B) 0.5 cyc/oct, and (C) 1.0 cyc/oct. The bottom row shows scores in thresholds (dB) corresponding to 75% correct for (D) 0.5 cyc/oct and (E) 1.0 cyc/oct. Significant pair‐wise comparisons are denoted by the brackets and the significance level (< 0.001) is denoted by ***. [Color figure can be viewed in the online issue, which is available at www.laryngoscope.com]

EasyQSMD thresholds significantly improved from activation to 1‐week post‐activation, then stabilized across subsequent timepoints. For clinical reference, EasyQSMD scores in percent correct across timepoints are shown in Figure 1A. For 0.5 cyc/oct (Figure 1), median thresholds decreased from 17.0 dB at activation to 14.2 dB at 1‐week, then stabilized (1‐month: 14.3 dB, 3‐months: 14.4 dB, 6‐months: 14.3 dB, and 1‐year: 13.1 dB). Mixed‐effects regression confirmed a significant main effect of timepoint (*F*(5, 722) = 49.40, *p* < 0.001, Figure 1B (percent correct) and Figure 1D (dB threshold)). Pairwise comparisons showed significant improvements between activation and all subsequent timepoints (*p* < 0.001), with no significant differences among post‐activation timepoints (*p* > 0.05), indicating that performance stabilized after the initial improvement from activation to 1‐week.

Similar patterns were observed for 1.0 cyc/oct thresholds (Figure 1), which decreased from 18.7 dB at activation to 16.2 dB at 1‐week, then stabilized through (1‐month: 15.8 dB, 3‐months: 15.7 dB, 6‐months: 15.7 dB, and 1‐year: 15.0 dB). Statistical analysis confirmed a significant main effect of timepoint (*F*(5, 646) = 31.25, *p* < 0.001, Figure 1C (percent correct) and Figure 1E (dB threshold)), with improvements occurring only between activation and all subsequent timepoints (*p* < 0.001), and no significant differences among the post‐activation measurements (*p* > 0.05) (Figure S1).

### 3.2 Relationship Between Early EasyQSMD Thresholds and Later Speech Recognition Scores

**FIGURE 2:**
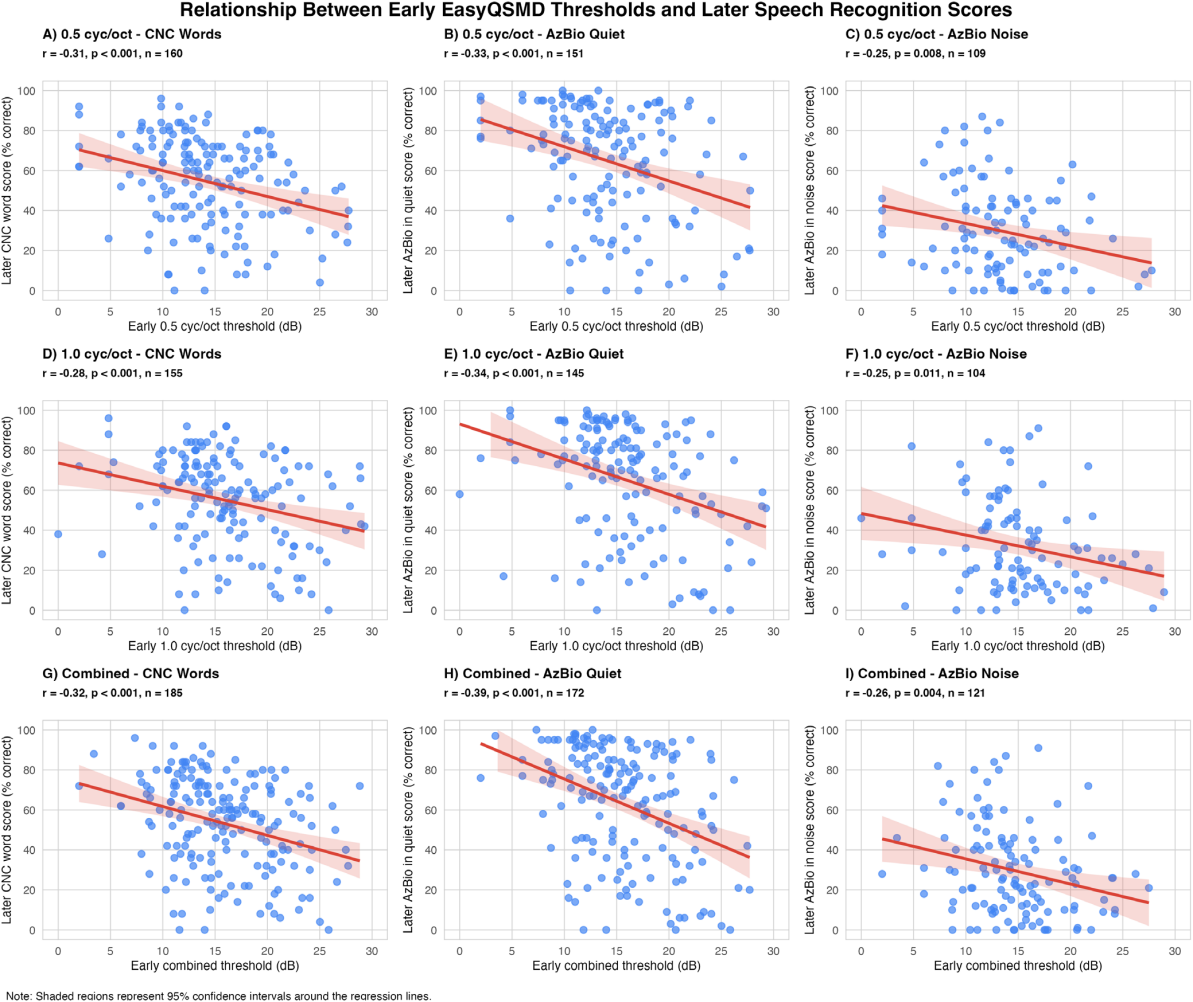
Pearson correlations between early EasyQSMD thresholds (model‐derived thresholds at 1‐month post‐activation) and later speech recognition scores (best of 6‐month and 1‐year). Panels A–C show 0.5 cyc/oct thresholds; panels D–F show 1.0 cyc/oct thresholds; panels G–I show combined thresholds (average of 0.5 and 1.0 cyc/oct). Panels A, D, and G show CNC word scores; panels B, E, and H show AzBio sentences in quiet; panels C, F, and I show AzBio sentences in noise. The red shaded regions represent the 95% confidence intervals around the regression lines. R^2^, *p* values, and sample sizes are shown in each panel. Note that lower thresholds (x‐axis) indicate better spectral resolution, while higher speech scores (y‐axis) indicate better performance. [Color figure can be viewed in the online issue, which is available at www.laryngoscope.com]

Growth curve model‐derived early EasyQSMD thresholds (predicted values at 1‐month post‐activation) showed significant associations with later speech recognition scores (best of 6‐months and 1‐year) across most measures (Figure 2). For 0.5 cyc/oct thresholds, significant negative associations were found with CNC word scores (*r* = −0.27, *p* = 0.002, *n* = 133, Figure 2A), AzBio sentences in quiet (*r* = −0.23, *p* = 0.008, *n* = 133, Figure 2B), and in noise (*r* = −0.28, *p* = 0.001, *n* = 133, Figure 2C). For 1.0 cyc/oct thresholds, a significant negative association was found with AzBio sentences in noise (*r* = −0.23, *p* = 0.010, *n* = 127, Figure 2F), while associations with CNC word scores (*r* = −0.17, *p* = 0.052, *n* = 127, Figure 2D) and AzBio sentences in quiet (*r* = −0.17, *p* = 0.052, *n* = 127, Figure 2E) approached but did not reach statistical significance. For combined thresholds (average of 0.5 and 1.0 cyc/oct), significant negative associations were observed with CNC word scores (*r* = −0.27, *p* = 0.002, *n* = 127, Figure 2G), AzBio sentences in quiet (*r* = −0.25, *p* = 0.005, *n* = 127, Figure 2H), and AzBio sentences in noise (*r* = −0.29, *p* = 0.001, *n* = 127, Figure 2I). These negative associations indicate that better spectral resolution (i.e., lower thresholds) relates to better speech recognition. Individual speech recognition trajectories showed substantial variability across participants (Figure S2).

### 3.3 Predictive Value of Early EasyQSMD Thresholds for Later Speech Recognition

Linear regression analyses confirmed that model‐derived early EasyQSMD thresholds significantly predicted later speech recognition outcomes across most measures, as shown in Table 2. For CNC word scores, model‐derived thresholds were significant predictors using combined thresholds (*R* ^2^ = 0.07, *β* = −1.99, *p* = 0.002, 95% CI: [−3.25, −0.73], *n* = 127), and 0.5 cyc/oct thresholds (*R* ^2^ = 0.07, *β* = −1.58, *p* = 0.002, 95% CI: [−2.58, −0.59], *n* = 133), while 1.0 cyc/oct thresholds approached but did not reach significance (*R* ^2^ = 0.03, *β* = −1.34, *p* = 0.052, 95% CI: [−2.70, 0.01], *n* = 127). The regression model using combined thresholds (CNC words = 76.0–1.99 × threshold) indicates that for every 1 dB improvement in the model‐derived early threshold, later CNC word scores increased by approximately 2.0 percentage points. For AzBio sentences in quiet, the predictive relationships were similar: combined thresholds (*R* ^2^ = 0.06, *β* = −2.16, *p* = 0.005, 95% CI: [−3.64, −0.68], *n* = 127), 0.5 cyc/oct thresholds (*R* ^2^ = 0.05, *β* = −1.59, *p* = 0.008, 95% CI: [−2.76, −0.43], *n* = 133), while 1.0 cyc/oct thresholds approached significance (*R* ^2^ = 0.03, *β* = −1.57, *p* = 0.052, 95% CI: [−3.15, 0.01], *n* = 127). For AzBio sentences in noise, model‐derived thresholds significantly predicted outcomes across all three threshold measures: combined thresholds (*R* ^2^ = 0.08, *β* = −2.47, *p* = 0.001, 95% CI: [−3.94, −1.01], *n* = 127), 0.5 cyc/oct thresholds (*R* ^2^ = 0.08, *β* = −1.89, *p* = 0.001, 95% CI: [−3.02, −0.76], *n* = 133), and 1.0 cyc/oct thresholds (*R* ^2^ = 0.05, *β* = −2.08, *p* = 0.010, 95% CI: [−3.64, −0.52], *n* = 127). The predictive relationship was somewhat stronger for AzBio sentences in noise (*R* ^2^ = 0.05–0.08) and CNC words (*R* ^2^ = 0.03–0.07) compared to AzBio sentences in quiet (*R* ^2^ = 0.03–0.06), though it should be noted all of these *R* ^2^ values across measures are modest.

**TABLE 2:** Predictive value of model‐derived early EasyQSMD thresholds for later speech recognition.

Predictor	Speech measure	*n*	*R* ^2^	β	95% CI	*p*
0.5 cyc/oct threshold	CNC words	133	0.07	−1.58	[−2.58, −0.59]	0.002
0.5 cyc/oct threshold	AzBio quiet	133	0.05	−1.58	[−2.76, −0.43]	0.008
0.5 cyc/oct threshold	AzBio noise	133	0.08	−1.89	[−3.02, −0.76]	0.001
1.0 cyc/oct threshold	CNC words	127	0.03	−1.34	[−2.70, 0.01]	0.052
1.0 cyc/oct threshold	AzBio quiet	127	0.03	−1.57	[−3.15, 0.01]	0.052
1.0 cyc/oct threshold	AzBio noise	127	0.05	−2.08	[−3.64, −0.52]	0.010
Combined threshold	CNC words	127	0.07	−1.99	[−3.25, −0.73]	0.002
Combined threshold	AzBio quiet	127	0.06	−2.16	[−3.64, −0.68]	0.005
Combined threshold	AzBio noise	127	0.08	−2.47	[−3.94, −1.01]	0.001

*Note*: Linear regression analyses examining the relationship between growth curve model‐derived EasyQSMD thresholds at 1‐month post‐activation and later speech recognition outcomes (best of 6‐months and 1 year). Combined threshold represents the average of 0.5 and 1.0 cyc/oct thresholds. Negative *β* values indicate that better spectral resolution (lower thresholds) predicts better speech recognition. Model‐derived thresholds were obtained from growth curve models with random intercepts and slopes using all available data from activation through 1‐year post‐activation.

Abbreviations: CI = confidence interval; cyc/oct = cycles/octave; *R* ^2^ = coefficient of determination; *β* = unstandardized regression coefficient representing the change in speech recognition score (% correct) per 1 dB improvement in spectral modulation detection threshold.

Supplementary analyses examined whether controlling for available demographic factors (age at implantation and biological sex) altered the predictive relationships. The addition of these covariates did not substantially change the predictive value of early EasyQSMD thresholds (changes in *R* ^2^ < 0.01), and neither covariate was a significant predictor when controlling for early spectral resolution (*p* > 0.15), suggesting that the relationship between early spectral resolution and later speech recognition is robust to these demographic factors.

## 4 Discussion

This investigation revealed that SMD, as measured by the EasyQSMD, stabilizes within 1 week post‐activation, aligning with prior research [31] while extending these findings across a substantially larger cohort (531 ears) with more frequent assessment timepoints. This rapid stabilization contrasts with the more gradual development of speech recognition documented in longitudinal CI outcome studies [12, 13, 14], supporting our hypothesis that spectral resolution abilities mature earlier than higher‐level linguistic skills—consistent with models positioning spectral resolution as a prerequisite for speech understanding [17].

Our analyses demonstrated significant associations between early EasyQSMD thresholds and later speech outcomes across most measures, with coefficients ranging from −0.25 to −0.39. These negative associations consistently indicate that better spectral resolution (lower thresholds) predicts better subsequent speech recognition. An important extension of our work was the inclusion of sentence recognition in both quiet and noise conditions, whereas previous research focused on word recognition [18, 19, 20, 21, 22, 23]. Finding significant associations across all three speech measures demonstrated that early spectral resolution abilities predicted performance across varying levels of linguistic and cognitive complexity. This provided a more comprehensive assessment of real‐world listening capabilities and underscored the fundamental importance of spectral resolution across different levels of speech processing.

The predictive relationship between early spectral resolution and later speech recognition outcomes likely operates through multiple mechanistic pathways. First, spectral resolution provides the foundational ability to discriminate between speech sounds with similar temporal but different spectral characteristics, such as vowels and nasals [18, 19, 20, 21, 22, 23]. Better spectral resolution early after activation may enable more accurate phoneme perception, which then facilitates more efficient top‐down linguistic processing and vocabulary access during speech recognition tasks [24, 25, 26, 27, 28]. Second, spectral resolution may reflect the quality of the electrode‐neural interface, including factors such as neural survival patterns, electrode placement, and current spread [33, 34, 35, 36]. Recipients with better early spectral resolution may have more favorable cochlear anatomy and neural survival, which would be expected to support better long‐term outcomes. Third, early spectral resolution may indicate how effectively the auditory system adapts to the novel electrical stimulation pattern provided by the CI. Recipients who demonstrate good spectral resolution may have greater neural plasticity or more efficient perceptual learning mechanisms, enabling them to better exploit the spectral information available through their device over time [17].

Importantly, these mechanisms are not mutually exclusive and likely interact in complex ways. The modest *R* ^2^ values we observed (3%–8% of the variance explained) reflect this multifactorial nature, suggesting that while early spectral resolution provides important predictive information, other factors—including cognitive abilities, duration of deafness, device usage patterns, and electrode characteristics—also substantially contribute to long‐term speech recognition outcomes.

The relationship between early spectral resolution and later speech recognition is likely mediated by cognitive factors including working memory, attention, and inhibition‐concentration abilities [15, 16]. Recipients with stronger cognitive abilities may be better able to leverage spectral information to support phoneme identification and may more effectively use top‐down processing to compensate when spectral resolution is limited [24, 25, 26, 27, 28]. Conversely, recipients with weaker cognitive abilities may struggle to compensate for poor spectral resolution, potentially explaining some of the variability in outcomes we observed. Unfortunately, our retrospective dataset did not include cognitive assessments, preventing us from examining these relationships directly. Future prospective research should incorporate validated cognitive measures alongside spectral resolution and speech recognition assessments to clarify how cognitive factors mediate the pathway from bottom‐up sensory processing to functional speech understanding.

Linear regression analyses quantified the predictive relationship between early spectral resolution and later speech recognition outcomes. Early EasyQSMD thresholds accounted for 3%–8% of variance in speech recognition performance, with the strongest relationship observed for AzBio sentences in noise (*R* ^2^ = 0.08). In practical terms, each 1 dB improvement in early threshold corresponded to a 1.3–2.5 percentage point increase in later speech recognition scores. While these *R* ^2^ values are modest and imply that other factors also contribute substantially to outcomes, the consistent significance of these relationships across most measures demonstrates meaningful clinical utility for early spectral resolution assessment in identifying potential individual performance trajectories.

The early stabilization of spectral resolution creates a valuable opportunity for clinicians. By administering the EasyQSMD during the first post‐activation month, potential poor performers could be identified before speech recognition skills fully develop, providing an opportunity for earlier implementation of targeted interventions such as re‐programming, auditory training, or reviewing expectations. This approach addresses a fundamental gap in current clinical practice, where poor performers often remain unidentified until months after activation, potentially leading to dissatisfaction, regret, or non‐use.

The modest yet significant predictive power of early EasyQSMD thresholds reflects the complex, multifactorial nature of CI outcomes. While spectral resolution provides an important foundation for speech recognition, other factors including cognitive abilities, age, duration of deafness, device wear time, electrode type and positioning also contribute to variability in outcomes [15, 16, 37, 38, 39]. Future research should explore multivariate predictive models that incorporate these additional factors alongside spectral resolution measures to improve prognostic accuracy. Given the findings that different threshold measures (0.5 cyc/oct, 1.0 cyc/oct, and combined) showed varying predictive strengths for different speech recognition tasks, further investigation is warranted to determine an optimized test protocol to integrate the EasyQSMD into busy clinics.

An additional consideration is that the relationship between spectral resolution and speech recognition may not be strictly linear across the full range of performance. Visual inspection of scatter plots in Figure 2 suggests potential non‐linearities, particularly at the extremes of performance. While exploratory quadratic regression models did not significantly improve model fit beyond linear models in our data, this may reflect limited statistical power given our sample sizes rather than true linearity. It is plausible that there are floor and ceiling effects, where improvements in spectral resolution have diminishing returns at very good or very poor performance levels. Future research with larger samples should examine potential non‐linear relationships, including threshold effects or interactions with other factors, which could inform identification of recipients most likely to benefit from interventions targeting spectral resolution.

While the EasyQSMD demonstrated significant predictive value, other spectral resolution measures may provide complementary information. Spectral‐temporally modulated ripple tests (SMRT) [40], pitch ranking tasks [41], and psychophysical tuning curves (PTCs) [42] assess related but distinct aspects of spectral processing that may capture different dimensions of auditory function. Future research should compare the predictive validity of these various measures and investigate whether combining multiple spectral resolution metrics in a composite score might improve prediction accuracy. Additionally, the EasyQSMD could potentially be enhanced by including additional modulation rates or by incorporating adaptive testing procedures that more efficiently identify each individual's threshold, which could improve precision while reducing administration time.

Our study benefited from a large sample, enhancing generalizability, but several limitations should be noted. First, our dataset lacked comprehensive demographic and audiological information due to its retrospective nature. While we examined available demographic factors (age at implantation and biological sex) and found that they did not substantially alter the predictive relationships, we lacked systematic information on duration of hearing loss, etiology, cognitive abilities, and listening configuration. These unmeasured variables likely contribute to the modest *R* ^2^ values observed and represent important directions for future research with prospectively collected data. Second, the absence of pre‐test training may have introduced learning effects contributing to the activation‐to‐1‐week improvement, though this mirrors real‐world clinical implementation. Third, the fixed testing order (EasyQSMD administered last) may have introduced fatigue effects, particularly at later timepoints when the full test battery was longest. If fatigue systematically reduced EasyQSMD performance, this could have weakened the observed relationships, suggesting our findings may represent conservative estimates of the true predictive values. Fourth, regarding missing data, we examined whether participants with complete versus incomplete data differed on baseline characteristics and found no significant differences, supporting the missing‐at‐random assumption. However, if participants with poorer outcomes systemically dropped out for unmeasured reasons beyond baseline performance, our results may underestimate the true variability in trajectories. Finally, all participants were postlingually deafened adults, limiting generalizability to prelingually deafened individuals or children, who may show different patterns of spectral resolution development and different relationships with speech outcomes.

## 5 Conclusion

SMD, as measured by the EasyQSMD, stabilizes within 1 week post‐activation and significantly predicts later speech recognition outcomes in postlingually deafened adult CI recipients. Model‐derived early spectral resolution thresholds at 1‐month accounted for 6%–15% of variance in later speech recognition performance, with each 1 dB improvement corresponding to 1.3–2.5 percentage point increases in speech scores. This early stabilization creates a critical clinical opportunity: recipients with poor spectral resolution can be identified during the first month, enabling earlier implementation of targeted interventions. While the modest predictive power reflects the multifactorial nature of CI outcomes, early spectral resolution provides valuable prognostic information during the critical early rehabilitation period. The EasyQSMD offers practical advantages for clinical implementation: it is non‐linguistic, requires less than 5 min to administer, and demonstrates significant predictive validity for long‐term outcomes. These characteristics make it particularly valuable for multilingual populations and as a complement to traditional speech recognition measures. Integrating spectral resolution assessment into early post‐activation clinical protocols could improve individualized expectations counseling and guide targeted interventions.

## Funding

This project was supported by award numbers R01 DC13117 and R01 DC009404 (PI: Gifford) from the National Institutes of Health.

## Conflicts of Interest

R.H.G. is on the audiology advisory boards for Advanced Bionics and Cochlear Americas and is a consultant for Skylark Bio. The other authors declare no conflicts of interest.

## Supporting information


**Figure S1:** Individual EasyQSMD threshold trajectories rank‐ordered by final performance. (A) EasyQSMD thresholds at 0.5 cyc/oct measured at activation, 1‐week, 1‐month, 3‐months, 6‐months, and 1‐year post‐activation. (B) EasyQSMD thresholds at 1.0 cyc/oct measured at activation, 1‐week, 1‐month, 3‐months, 6‐months, and 1‐year post‐activation. Each thin line represents an individual participant's trajectory. Thick black lines represent group median, with error bars showing interquartile ranges. Lower thresholds (in dB) indicate better spectral resolution. Trajectories are arranged from highest (top) to lowest (bottom) final thresholds, demonstrating rapid stabilization after the initial improvement from activation to 1‐week alongside considerable individual variability.

**Figure S2:** Individual speech recognition trajectories rank‐ordered by final performance. (A) CNC word recognition scores at 1‐month, 3‐months, 6‐months, and 1‐year post‐activation. (B) AzBio sentence recognition in quiet at 1‐month, 3‐months, 6‐months, and 1‐year post‐activation. (C) AzBio sentence recognition in +5 dB SNR noise at 3‐months, 6‐months, and 1‐year post‐activation. Each thin red line represents an individual participant's trajectory. Thick black lines represent group median, with error bars showing interquartile ranges. Higher scores indicate better performance. Trajectories are arranged from lowest (bottom) to highest (top) final performance, illustrating substantial individual variability alongside overall patterns of improvement.

## Data Availability

The data that support the findings of this study are available from the corresponding author upon reasonable request.
