# Supplementary figures and images for: Early Spectral Resolution Predicts Later Speech Recognition in Adult Cochlear Implant Recipients
Source: Laryngoscope. 2026 Feb 23;136(7):3155–64. doi: 10.1002/lary.70383 (PMC13253172; doi:10.1002/lary.70383)

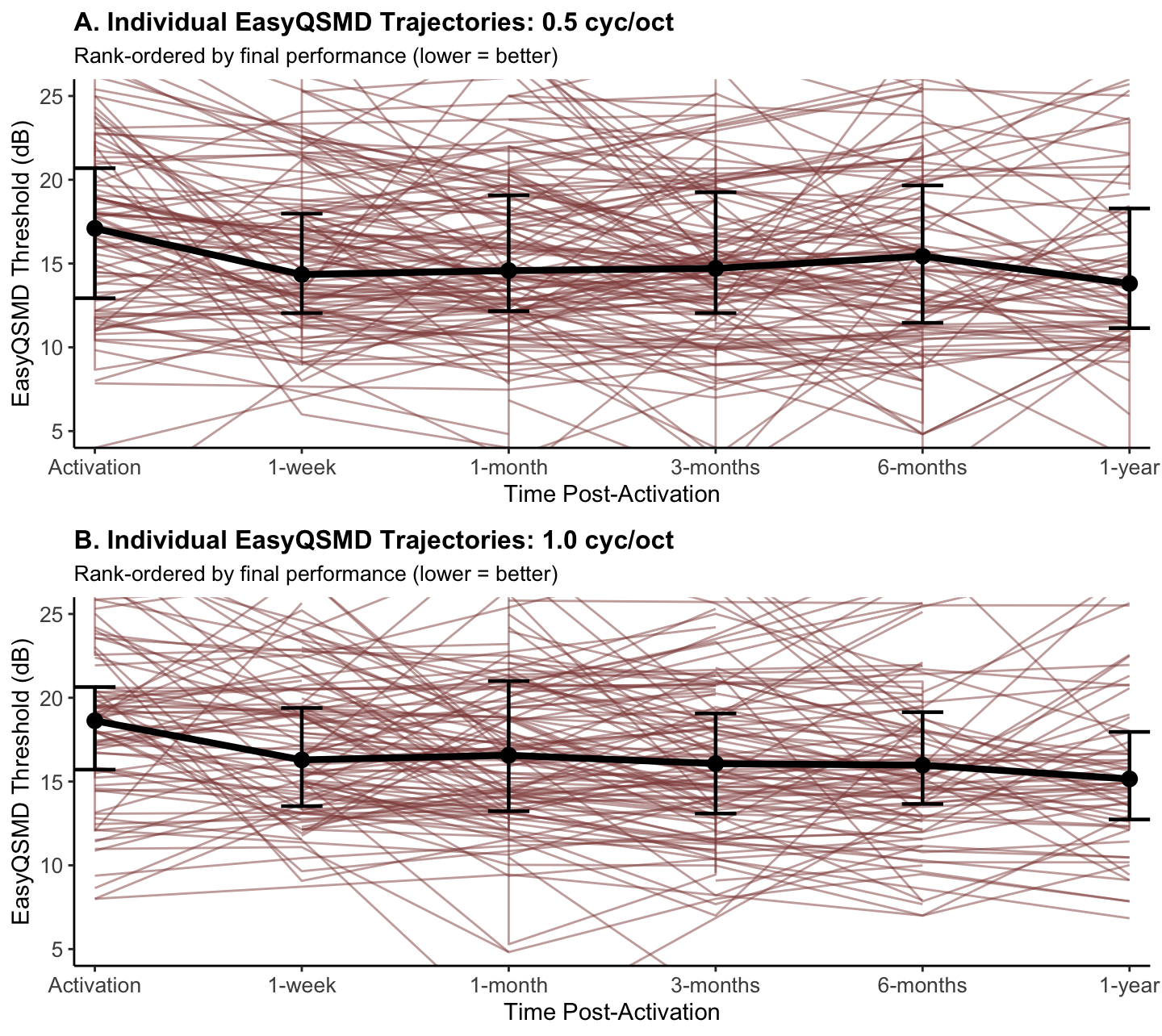

Supplement: Supplementary file 1 — Figure S1: Individual EasyQSMD threshold trajectories rank‐ordered by final performance. (A) EasyQSMD thresholds at 0.5 cyc/oct measured at activation, 1‐week, 1‐month, 3‐months, 6‐months, and 1‐year post‐activation. (B) EasyQSMD thresholds at 1.0 cyc/oct measured at activation, 1‐week, 1‐month, 3‐months, 6‐months, and 1‐year post‐activation. Each thin line represents an individual participant's trajectory. Thick black lines represent group median, with error bars showing interquartile ranges. Lower thresholds (in dB) indicate better spectral resolution. Trajectories are arranged from highest (top) to lowest (bottom) final thresholds, demonstrating rapid stabilization after the initial improvement from activation to 1‐week alongside considerable individual variability. [file LARY-136-3155-s001.png]

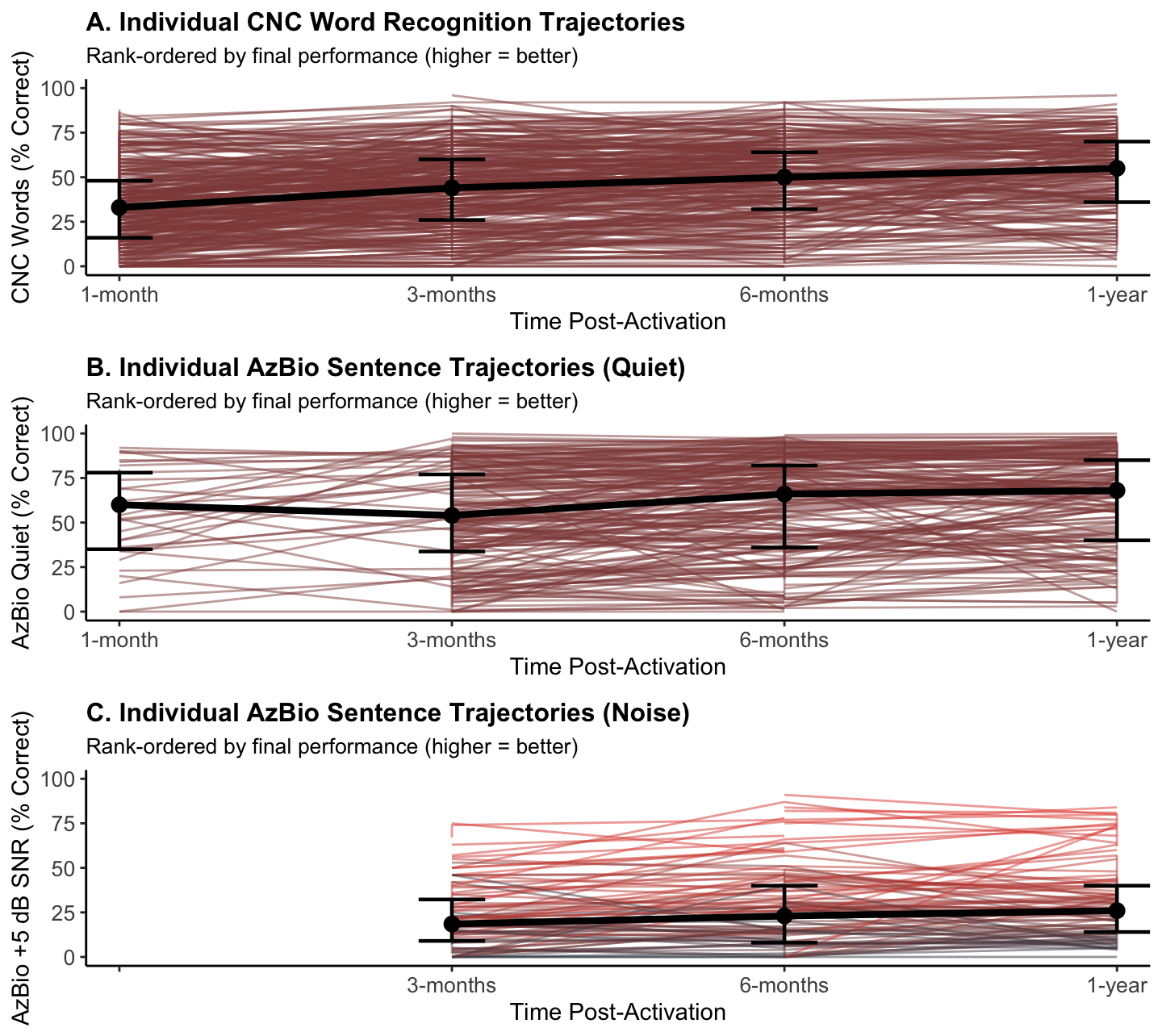

Supplement: Supplementary file 2 — Figure S2: Individual speech recognition trajectories rank‐ordered by final performance. (A) CNC word recognition scores at 1‐month, 3‐months, 6‐months, and 1‐year post‐activation. (B) AzBio sentence recognition in quiet at 1‐month, 3‐months, 6‐months, and 1‐year post‐activation. (C) AzBio sentence recognition in +5 dB SNR noise at 3‐months, 6‐months, and 1‐year post‐activation. Each thin red line represents an individual participant's trajectory. Thick black lines represent group median, with error bars showing interquartile ranges. Higher scores indicate better performance. Trajectories are arranged from lowest (bottom) to highest (top) final performance, illustrating substantial individual variability alongside overall patterns of improvement. [file LARY-136-3155-s002.png]
